# Leveraging soil microbiome diversity for the management of highly virulent *Fusarium* wilt (FOV4) in cotton

**DOI:** 10.3389/fmicb.2026.1856023

**Published:** 2026-07-31

**Authors:** Ilham Laadsi, Mostafa Abdelrahman, Mauricio Ulloa, Mohamed Fokar, Timothy O. Jobe

**Affiliations:** 1 Center for Biotechnology and Genomics, Texas Tech University, Lubbock, TX, United States; 2 Institute for One Health and Innovation, Texas Tech University, Lubbock, TX, United States; 3 Cropping Systems Research Lab, Plant Stress and Germplasm Development Research, United States Department of Agriculture–Agricultural Research Service, Plains Area, Lubbock, TX, United States

**Keywords:** cotton, fungal biome, *Fusarium oxysporum* f. sp. *vasinfectum*, *Fusarium* wilt, soil microbiome

## Abstract

**Introduction:**

Cotton (*Gossypium* spp.) is a globally important crop increasingly threatened by *Fusarium oxysporum* f. sp. *vasinfectum* race 4 (FOV4), a soil-borne pathogen responsible for Fusarium wilt. FOV4 has negatively affected cotton production in California and was confirmed in the far west Texas region of El Paso, TX in 2017, where it has caused similar disruptions. Thus, there is an urgent need for improved disease management and the development of resistant commercial cotton cultivars to maintain agricultural productivity.

**Methods:**

To understand the relationships among soil properties, fungal communities, and disease incidence, we examined the elemental composition and fungal microbiome of five cotton fields in the lower valley of El Paso, Texas region, having varying levels of Fusarium wilt incidence. Comparisons between high Fusarium wilt incidence fields (F1, F2, and F5) and low Fusarium wilt incidence fields (F3 and F4) were performed. Metabarcoding analyses identified marked differences in fungal community composition and diversity between the fields.

**Results:**

Alpha diversity metrics indicated higher fungal diversity and evenness in the low Fusarium wilt incidence field F4, suggesting that high fungal diversity contributes to decreased disease incidence. In contrast, high Fusarium wilt incidence fields (F1, F2, and F5) exhibited lower diversity, indicative of a less resilient fungal ecosystem. Beta diversity analyses further confirmed the distinct fungal community composition between soils with contrasting Fusarium wilt incidence. Taxonomic profiling showed that the low Fusarium wilt incidence field F4 harbored beneficial fungal taxa, including *Actinomucor, Fusarium* (potentially non-pathogenic species), *Penicillium, Preussia*, and *Pseudeurotium*, generally recognized for their contributions to soil health and potential to suppress pathogenic organisms. In contrast, the high Fusarium wilt incidence fields were dominated by genera associated with plant pathogenicity, such as *Alternaria*, *Cladosporium*, and *Stachybotrys*, contributing to the higher disease prevalence observed.

**Discussion:**

These findings underscore the crucial role of fungal diversity and soil chemical composition in influencing the incidence of Fusarium wilt in cotton. Soils with low Fusarium wilt incidence, characterized by diverse and complex fungal communities, may suppress the establishment and proliferation of pathogens. Our results provide insights for developing targeted soil management practices and enhancing cotton resilience, essential for developing sustainable disease management strategies and breeding resistant cultivars.

## 1 Background

In natural environments, healthy and asymptomatic plants coexist with a diverse array of microorganisms, including archaea, bacteria, fungi, and protists, collectively referred to as the plant microbiota. These microbes form intricate microbial consortia that significantly influence plant growth and productivity, enhance nutrient acquisition, increase stress tolerance, and strengthen resistance to pathogens (Hassani et al., 2018). Although plants possess mechanisms to cope with biotic and abiotic stresses, they rely heavily on their microbiota for survival and defense against these stresses (Turner et al., 2013).

Environmental stressors and shifts in temperature, precipitation, and extreme weather events can significantly alter the composition and activity of plant microbial communities. These environmental challenges directly influence the diversity and abundance of microbial populations associated with plants, potentially impacting plant health, resilience, and overall ecosystem functionality (In 't Zandt et al., 2025). As environmental conditions evolve, microbial communities residing within and on plants also adapt, resulting in outcomes that can be either beneficial or detrimental. As temperature, humidity, and precipitation patterns fluctuate, the dynamics of microbial communities also change, increasing the potential for outbreaks of various plant diseases, including those caused by fungi. Furthermore, as environmental conditions fluctuate, pathogens undergo evolutionary shifts to adapt to new environments, which can lead to increased virulence or the emergence of new diseases.

*Fusarium* wilt (FW) is caused by the soilborne fungal pathogen *Fusarium oxysporum*, which comprises over 140 formae speciales and affects a wide variety of crops across at least 45 plant families (Edel-Hermann and Lecomte, 2019). *Fusarium oxysporum* f. sp. *vasinfectum* (FOV) is the pathogen responsible for FW in cotton (*Gossypium* spp.) and is prevalent in cotton-producing areas worldwide. The National Cotton Council of America reported that FW/FOV has consistently caused significant economic damage in all cotton-producing states, with estimated yield losses of $15 million in 2021 (Crop Protection Network, 2022). Thus, FOV poses a significant threat to cotton production throughout the entire “Cotton Belt” of the U. S. and worldwide. So far, the best control to manage FOV4 is by planting resistant cultivars (Ulloa et al., 2006, 2016, 2022, 2023).

*Fusarium oxysporum* f. sp. *vasinfectum (FOV)* was first reported in 1892 from samples collected in Alabama and Arkansas (Atkinson, 1892). The typical symptoms of FW are leaf chlorosis and plant wilting due to xylem clogging, which results in characteristic vascular discoloration (Lawrence et al., 2018). Traditionally, FOV isolates have been classified into eight races (FOV1-FOV8) based on their pathogenicity and entry route. However, DNA sequence analysis, particularly of the translation elongation factor-1α (EF-1α) and rDNA intergenic spacer (IGS) regions, revealed that FOV3 and FOV5, as well as FOV4 and FOV7, are genetically indistinguishable. Thus, there are currently six races of FOV - FOV1, 2, 3/5, 6, 4/7, and 8–that differ in their pathogenicity and virulence to various cotton cultivars (Kim et al., 2005; Skovgaard et al., 2001). Additionally, unique genotypes have been identified in Australia, and new aggressive genotypes, such as LA108, LA110, LA127/140, and MDS-12, have emerged in the southeastern U. S. (Cianchetta et al., 2015; Holmes et al., 2009).

*Fusarium oxysporum* f. sp. *vasinfectum (FOV)* races 1, 3, and 8 exhibit mild virulence, causing minimal symptoms in cotton cultivars unless the plants are also infected with root-knot nematodes (Meloidogyne incognita). In contrast, FOV4 is notably more virulent and can cause significant plant mortality, even in the absence of root-knot nematodes. In the U. S., a belt-wide survey conducted from 2012 to 2013 revealed that races 1, 2, 3, and 8 were widely distributed across the region. However, race 4 was exclusively found in California at that time (Cianchetta et al., 2015). In the southwest region, which encompasses New Mexico and the far west Texas areas of El Paso and Hudspeth Counties, seedling diseases have historically posed a minimal issue for cotton production due to the extremely dry conditions (Zhu et al., 2020). However, in 2017, FOV4 was reported in New Mexico and Texas (Halpern et al., 2018), suggesting that changes in the disease complex or environment may have occurred, which in turn affected its prevalence (Ulloa et al., 2023). FOV4 differs from races 1 and 2 in its ability to cause symptoms even in the absence of root-knot nematodes and its capability to induce disease in alkaline soils (Kim et al., 2005). Thus, gaining insight into the soil composition and microbial community within cotton fields is crucial for managing FOV-infested fields, developing effective strategies to mitigate the spread of the disease, and breeding resistant cotton cultivars.

The density, diversity, and structure of the soil microbiome are major factors influencing disease suppression and incidence (Rangan and Hang, 2017). Beneficial microbial taxa in soil have been shown to control soil-borne diseases by directly inhibiting a range of pathogens (Todorović et al., 2023). These protective microbes function through several mechanisms, including the production of antimicrobial or toxic substances, competition for space and resources, and induction of plant-protective responses through systemic metabolic regulation of plant physiology and immune functions (Yuan et al., 2021). The impact of plant-protective soil microbiota is particularly significant in disease-suppressive soils, which are inhospitable to certain plant pathogens. In these soils, pathogens either fail to establish or persist, establish but cause minimal to no damage, or initially establish and cause disease that diminishes with continued monoculture of the crop (Baker and Cook, 1974). Disease-suppressive soils serve as reservoirs for beneficial microorganisms that can protect plants from various soil-borne diseases, including those caused by *Fusarium* spp. (Expósito et al., 2017). However, the influence of soil microbiome composition and ecology on FOV prevalence in cotton fields remains insufficiently understood. This study investigated the fungal diversity and soil properties of five fields in the far west Texas region of El Paso County, which demonstrated suppressive or conducive soil behaviors when cultivated with different cotton varieties or cultivars. The elemental composition of the five soils was analyzed to determine chemical differences across the fields. Additionally, metabarcoding analyses of bulk soil using the internal transcribed spacer (ITS) amplicon was performed to identify the composition and abundance of fungal taxa regulating FOV4 disease prevalence in cotton. These objectives facilitate monitoring and comparison of soil microbiomes and their impact on FOV disease dynamics. These findings will support the development of more effective strategies for cotton breeding and disease management, ultimately improving cotton yield and sustainability.

## 2 Methods

### 2.1 Preparation, sequencing, and library construction of ITS gene samples

A total of 20 rhizosphere soil samples were collected from five cotton fields, with a disease severity evaluated using vascular root staining (VRS) scores ranging from 0 (no staining) to 5 (plant death) in the El Paso area (31.7619° N and 106.4850° W), Texas (5 fields × 4 biological replicates), and subjected to microbial DNA extraction using the DNeasy PowerSoil Pro Kit (Qiagen, MD, US). The Internal transcript spacer (ITS) gene was amplified with ITS1-forward (5’-TAGAGGAAGTAAAAGTCGTAA-3′) and ITS1-reverse (5′-TTYRCTRCGTTCTTCATC-3′) primers, as well as ITS2-forward (5′- GATGAAGAACGYAGYRAA-3′) and ITS2-reverse (5′- TCCTCCGCTTATTGATATGC-3′) primers using PCR conditions of 3 min at 95 °C, followed by 35 cycles of 30 s at 95 °C, 30 s at 55 °C, 30 s at 72 °C, and a final extension at 72 °C for 5 min. The amplified PCR products were purified using 20 μL AMPure XP magnetic beads per sample (Beckman Coulter Life Sciences, CA, United States). Next, Illumina sequencing adaptors and indexes were attached to purified DNA from each sample using the Nextra XT index kit (Illumina, CA, United States), with PCR conditions of 95 °C for 3 min, followed by 8 cycles of 95 °C for 30 s, 55 °C for 30 s, 72 °C for 30 s, and a final extension at 72 °C for 5 min. The integrity and size of the DNA fragments in the library were assessed using a Fragment Analyzer (AATI, IO, United States). The effective concentration of the library was determined using qPCR. The quantified libraries were pooled and sequenced using the Illumina MiSeq platform at.

Center of Biotechnology and Genomics, Texas Tech University (Lubbock, TX).

### 2.2 ITS abundance, taxonomy, and statistical evaluation

The raw read quality and sequencing depth were assessed using the qiime tools peek function in QIIME2 (Caporaso et al., 2010). Data processing, including denoising, chimera removal, error modeling, and abundance estimation, was systematically performed using the DADA2 plugin in QIIME2. The taxonomy of each amplicon sequence variant (ASV) was assigned using the classifier generated from the UNIT^1^ fungi database. Next, sequence alignment was performed using the Qiime alignment mafft function, followed by the construction of an unrooted phylogenetic tree with the Qiime phylogeny fasttree. The phylogenetic tree was then rooted using Qiime phylogeny midpoint-root function for further analysis. After removing mitochondrial and chloroplast reads, abundance, taxonomy, metadata, and phylogenetic tree objects were merged for the statistical analysis. Alpha and beta diversity analyses and visualizations were performed using a range of packages, including “phyloseq,” “ggplot2,” and “pheatmap” in R.4.3.2. A false discovery rate-corrected *p*-value ≤ 0.05 was considered statistically significant. Alpha and beta diversity comparisons among and between different crops were conducted in R.4.3.2 using analysis of variance (ANOVA), the Kruskal-Wallis rank sum test, and permutational multivariate analysis of variance (PERMANOVA).

### 2.3 Elemental analysis of soil samples using inductively coupled plasma optical emission spectrometry

A total of 0.250 g of the dried and homogenized soil sample was accurately weighed and transferred to a Teflon digestion vessel. To achieve complete digestion of the soil matrix, a combination of concentrated acids and an oxidizing agent was added to the vessel in the following sequence: 5 mL of nitric acid, a strong oxidizing acid that aids in the breakdown of organic matter and dissolution of most metal oxides; 2 mL of hydrofluoric acid, essential for dissolving silicate minerals and refractory components present in soil samples; 2 mL of hydrochloric acid, which helps maintain metal solubility and prevents precipitation of certain elements; and finally, 1 mL of 30% hydrogen peroxide, acting as an oxidizing agent to enhance organic matter decomposition. Once the reagents were added, the digestion vessel was securely sealed and placed in a microwave digestion system. Digestion was performed under controlled conditions, maintaining a temperature range of 180–200 °C for a total duration of 40 min. After digestion, the vessels were allowed to cool to room temperature before being carefully opened inside a fume hood to prevent exposure to residual acid vapors. The digested solution was then diluted with deionized water to a final volume of 50 mL, filtered, and prepared for injection in an ICP-OES (5,110, Agilent, United States) (Grindlay et al., 2016). For calibration and standardization, a series of multi-element calibration standards were prepared at concentrations of 0.1, 1, 10, and 50 ppm to generate a standard curve for quantifying elemental concentrations across different soil samples. A blank solution (2% HNO₃) was used for baseline correction and to monitor instrument drift. The instrument quantified elemental concentrations based on a calibration curve generated from prepared multi-element standards. The detected concentrations were initially measured in parts per million in solution and subsequently converted to mg/kg in soil using the sample weight and the final dilution volume.

## 3 Results

### 3.1 Analysis of disease incidence and mortality rates across experimental conditions

**Figure 1:**
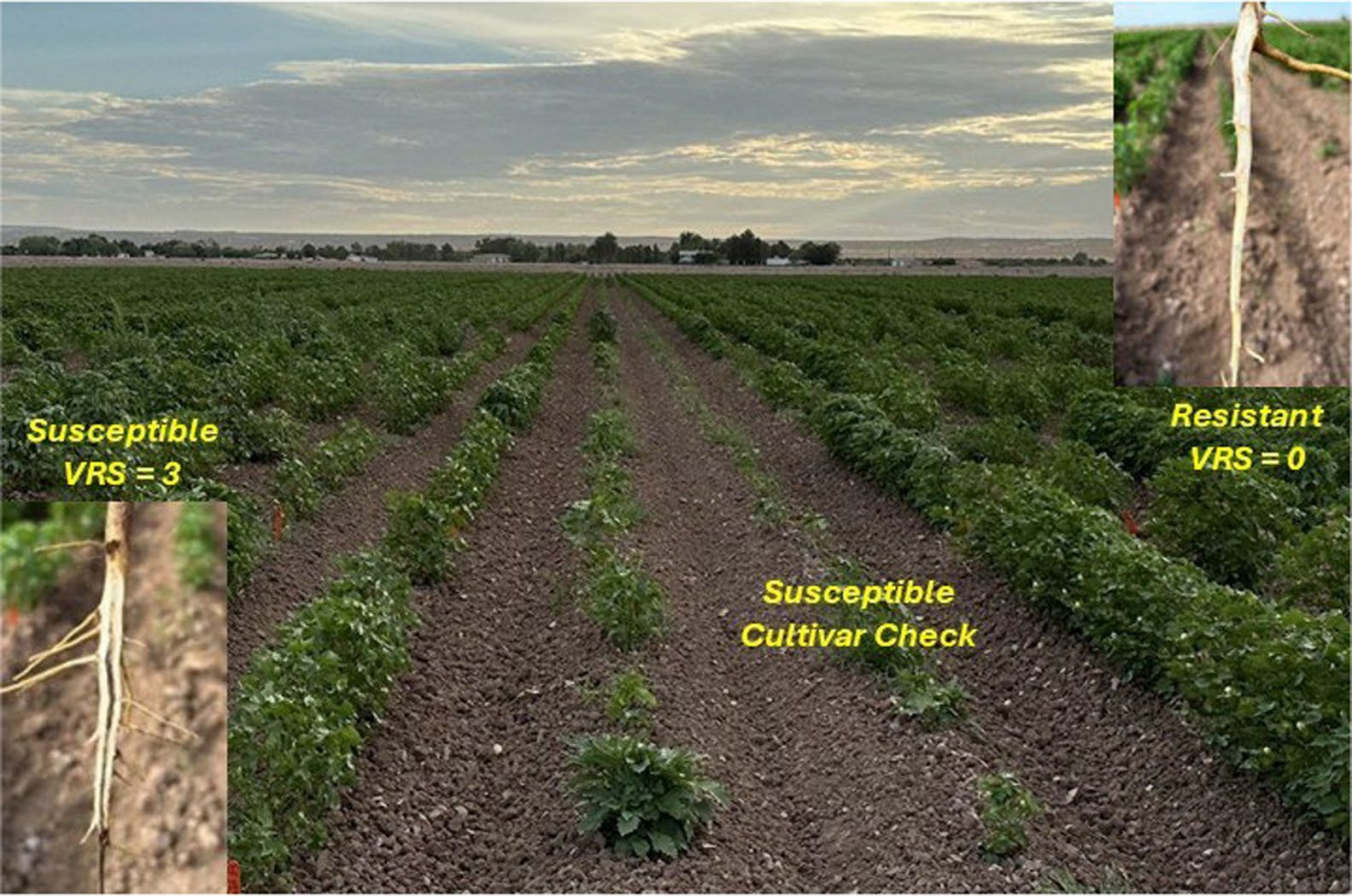
A naturally infested Fusarium wilt race 4 field in El Paso, TX region showing a susceptible cultivar check and symptomatic plants with vascular root staining (VRS) index, VRS ranged from 0 (no staining) to 5 (plant death).

Analysis of disease incidence and mortality rates across five cotton fields (F1–F5) during the 2023 and 2024 growing seasons revealed significant patterns associated with the presence of FOV4 (Table 1). Fields F1, F2, and F5 were confirmed to harbor FOV4, with disease severity assessed through percent mortality and vascular root staining (VRS) scores. In F1, mortality rates ranged from 30 to 45% in 2023 and decreased to 30–35% in 2024, with consistent VRS scores of 2–4, indicating moderate vascular damage and persistent pathogen presence. An example of a naturally infested FOV4 field with symptomatic plants is presented in Figure 1.

**Table 1:** Disease incidence and severity were assessed across five fields, including plant percent mortality and vascular root staining (VRS) scores.

	2023		2024	
Field	% Mortality	VRS	% Mortality	VRS
F1	30–45%	2–4	30–35%	2–4
F2	50–65%	2–4	45–60%	2–4
F3	0%	0	< 1%	NA
F4	0%	0	< 1%	NA
F5	10–25%	0–3	NA	NA

Fields 1 (F1), 2 (F2), and 5 (F5) were confirmed with *Fusarium* wilt [*Fusarium oxysporum* f. sp. *vasinfectum* (FOV)] Race 4 (FOV4). F1, F2, F3 2023, and 2024 values are based on overall observations of FOV4 susceptible Pima cotton spatially through the fields; 2023–2024 F3 Upland cotton; and 2023 F5 Pima FOV4 resistant cotton. The VRS ranged from 0 (no staining) to 5 (plant death). NA = no available data.

Field 2 exhibited the highest mortality, reaching 50–65% in 2023 and remaining at 45–60% in 2024, accompanied by similar VRS scores, which suggests a highly conducive environment for FOV4 proliferation. Field 5 displayed lower mortality rates (10–25%) in 2023, with VRS scores of 0–3, possibly due to the cultivation of Pima cotton varieties with partial resistance; data for 2024 were not available. In contrast, Fields 3 and 4 demonstrated suppressive characteristics, with F3 showing no mortality (0%) in 2023 and less than 1% in 2024, and F4 presenting similar trends across both seasons, indicating natural resistance potentially linked to the soil microbiota or resistant host varieties. Comparative analysis indicated that high-incidence fields (F1 and F2) shared susceptibility traits and environmental conditions conducive to FOV4 infection, whereas low-incidence fields (F3 and F4) suggested the presence of suppressive soil properties. Field F5 represents an intermediate case that may provide insights into partial resistance mechanisms. The VRS scores in F1 and F2, ranging from 2 to 4, underscore significant vascular colonization by FOV4 and correlate strongly with mortality rates. These findings emphasize the importance of investigating suppressive soil mechanisms in F3 and F4, examining genetic resistance in Pima cotton varieties observed in F5, and implementing pathogen monitoring and agronomic practices to mitigate FOV4 spread.

### 3.2 Alpha and beta diversity measurements of the bulk soil microbiomes from El Paso, TX region cotton fields demonstrate specificity to conducive and suppressive soil types

**Figure 2:**
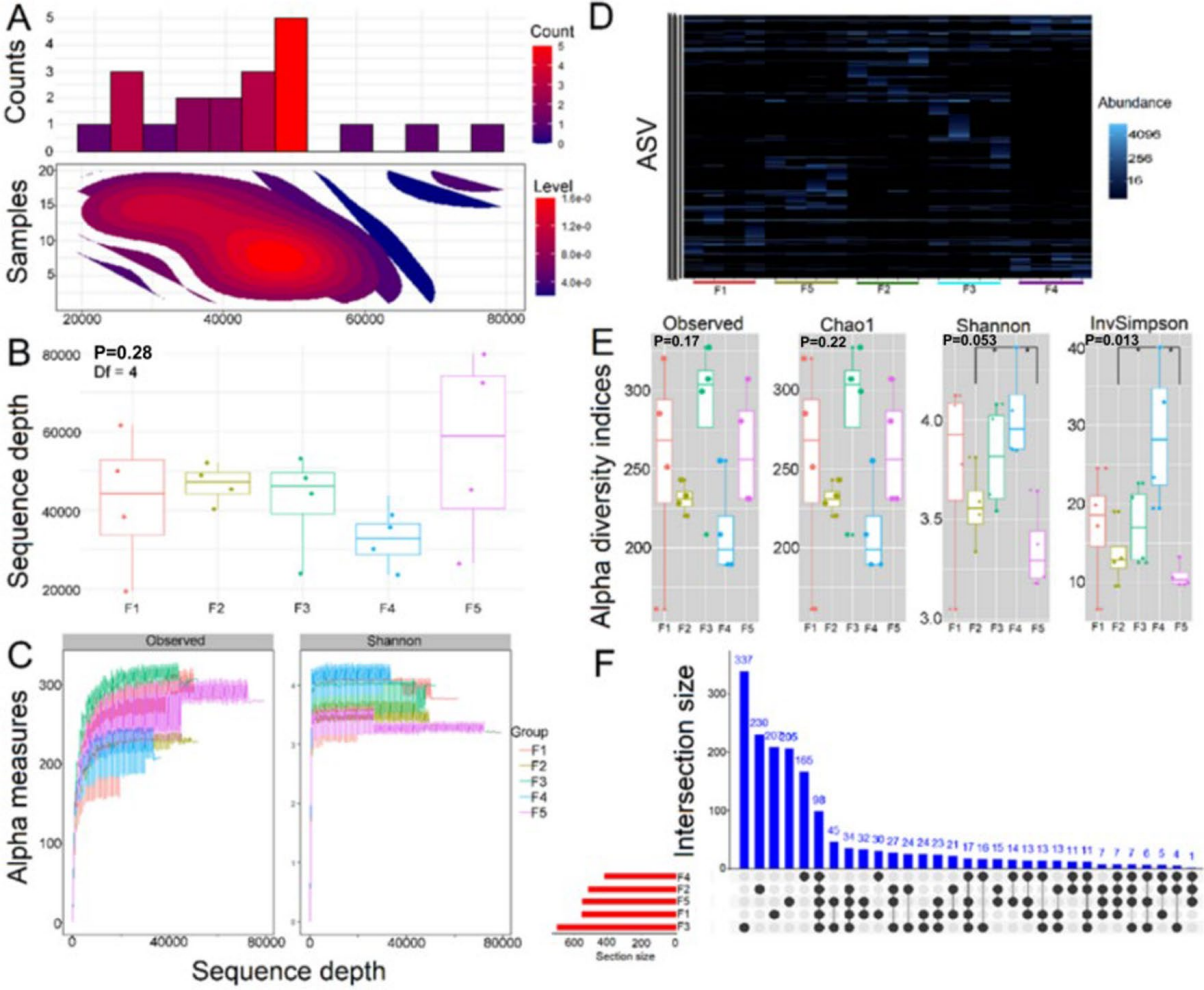
Alpha diversity and sequencing depth analysis in suppressive and conducive soils from the El Paso, TX region-fields. **(A)** Histogram and density plots representing the distribution of sample counts across different sequencing depths. **(B)** Boxplot displaying the sequencing depth across different soil groups (F1–F5). The *p*-value of 0.28 suggests that there was no statistically significant difference in sequencing depth among the groups, as determined by ANOVA. **(C)** Rarefaction curves illustrating the relationship between sequencing depth and alpha diversity measures, specifically observed species (left) and Shannon diversity index (right). Each curve represents a different soil group, F1–F5. **(D)** Heatmap visualizing the abundance of Amplicon Sequence Variants (ASVs) across different soil groups. The intensity of the color corresponds to the abundance level, with light-blue colors indicating higher abundance. **(E)** Boxplots of various alpha diversity indices (Observed, Chao1, Shannon, and Inverse Simpson) across soil groups F1–F5. The *p*-values indicate the statistical significance of the differences in diversity indices among the groups, as determined by the Kruskal-Wallis test. **(F)** Upset plot showing the intersection sizes of ASVs shared among the different soil groups. Vertical bars represent the number of ASVs present at the intersections of the groups indicated by the filled circles below. Horizontal bars on the left indicate the total number of ASVs in each group.

A panel of 20 soil samples collected from five cotton fields in the El Paso, TX region (31.7619° N, 106.4850° W) was analyzed to evaluate microbiome structure in conducive and suppressive bulk soils using internal transcribed spacer (ITS) amplicon-based metagenomics. The FOV4 status of these fields was previously reported (Diaz et al., 2021). Fields F3 and F4 exhibited low *Fusarium oxysporum* f. sp. *vasinfectum* race 4 (FOV4) incidence, while F1, F2, and F5 showed moderate to high incidence. Four biological replicates per field were collected to ensure robust and reproducible comparisons of soil fungal communities associated with varying *Fusarium* infection levels. Sequencing depth analysis indicated that most samples ranged from 20,000 to 50,000 reads, with the majority clustering around 40,000–50,000 reads (Figure 2A). No statistically significant differences in sequencing depth were observed among fields (ANOVA, *p* = 0.28; Figure 2B), confirming that differences in fungal diversity were not confounded by sequencing effort.

Rarefaction analysis for observed species and the Shannon diversity index demonstrated that diversity measures plateaued with increasing sequencing depth in all fields, indicating sufficient coverage to capture most fungal diversity (Figure 2C). Field F5 exhibited the highest amplicon sequence variant (ASV) richness, consistent with its greater sequencing depth. The heatmap of ASV abundance revealed clear differences between suppressive (F3 and F4) and conducive (F1, F2, F5) fields (Figure 2D). F5 displayed a higher number of abundant ASVs, aligning with its elevated diversity indices. ITS amplicon-based metagenome processing using the DADA2 method identified 1,662 ASVs across seven taxonomic ranks. Alpha diversity analysis using observed (*p* = 0.17) and Chao1 (*p* = 0.22) indices showed no significant differences in species richness among fields (Kruskal-Wallis test; Figure 2E). However, Shannon and Simpson diversity metrics revealed differences (*p* = 0.053 and *p* = 0.013, respectively), with the suppressive soil in F4 exhibiting higher fungal diversity than conducive soils in F2 and F5. These results highlight substantial variation in community structure, particularly in evenness, between fields with low and high FOV4 incidence. Upset plot analysis revealed that 98 ASVs were shared among all fields, while each field also contained a substantial number of unique ASVs, indicating distinct community structures potentially shaped by environmental or experimental factors. The limited overlap of ASVs between low and high FOV4 incidence soils underscores the specificity of microbiomes to their respective soil types.

**Figure 3:**
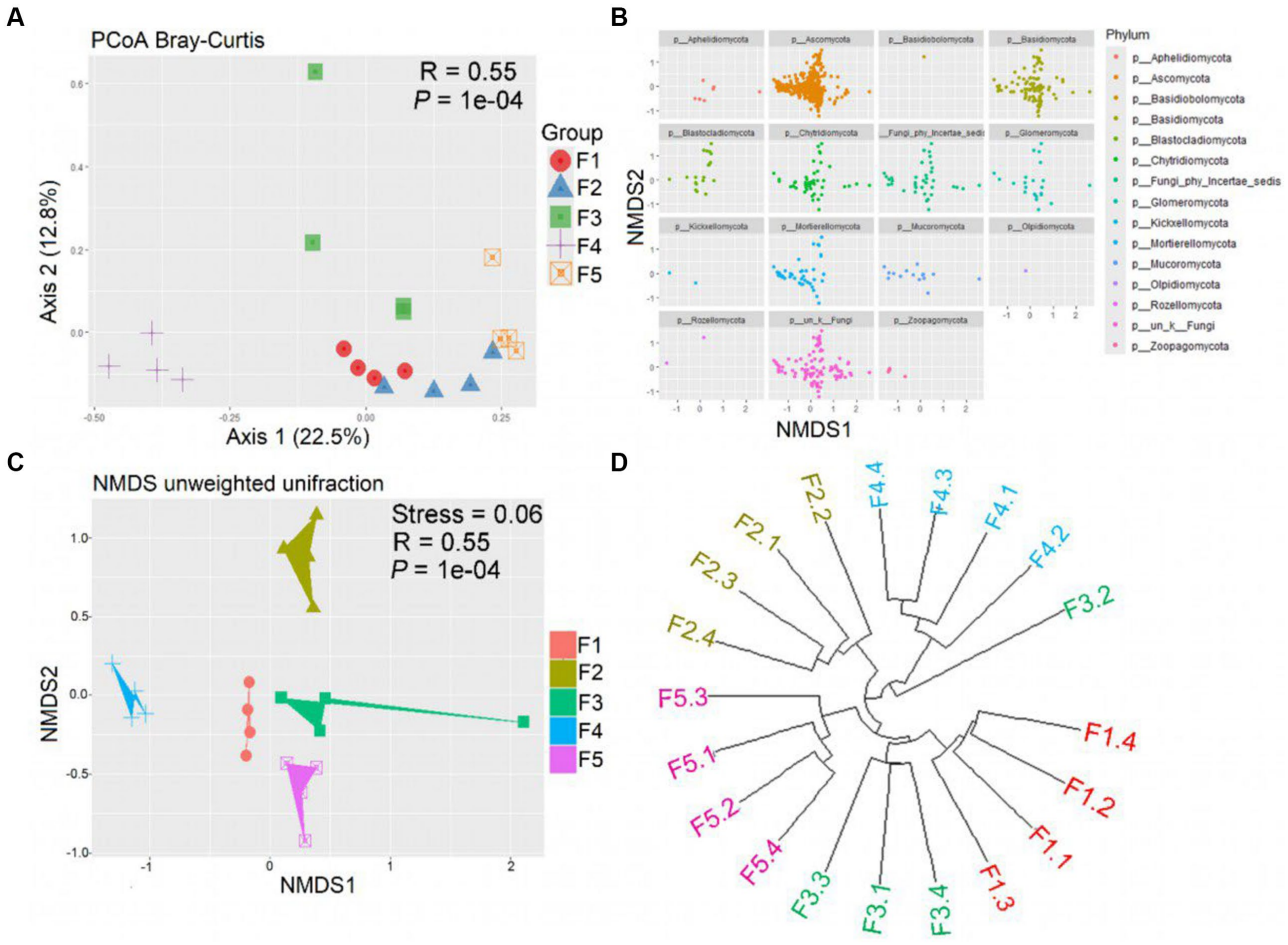
Multivariate analysis of fungal community composition across different fields from EL Paso, TX region. **(A)** Principal coordinate analysis (PCoA) based on Bray–Curtis dissimilarity metrics, showing the distribution of fungal communities across different fields (F1 to F5). Each point represents a technical replicate from the respective field. **(B)** Non-metric multidimensional scaling (NMDS) plot based on unweighted UniFrac distances, visualizing the variation in fungal communities among fields F1–F5. The stress value of 0.06 indicates a good representation of the data in the two-dimensional space. Similar to the PCoA plot, the groups are separated, with an R value of 0.55 and a *p*-value of 1e-04 confirming the significant distinction in community composition. **(C)** NMDS phylum-level composition of the fungal community, with each subplot representing a different fungal phylum. Points are colored according to phylum classification. **(D)** Hierarchical clustering of the investigated fields based on the abundance of their fungal communities. Technical replicates are indicated after the decimal in the numerical label (i.e., F1.1 is Field 1, replicate 1).

The beta-diversity was determined to measure the variation between soils with low and high FOV4 incidence (Figure 3). Both principal coordinate analysis (PCoA) based on Bray–Curtis dissimilarity metrics and non-metric multidimensional scaling (NMDS) based on unweighted UniFrac distances revealed significant differences in fungal communities between conducive and suppressive soils (Figures 2A,B). The PCoA analysis indicated a clear separation between the fungal communities of the two soil types, with an R value of 0.55, signifying a strong distinction in community composition. The two axes (Axis 1 and Axis 2) explain 22.5 and 12.8% of the variance, respectively, with most of the fields separated by Axis 2 (Figure 3A). Similarly, the NMDS analysis demonstrated significant differentiation, with a stress value of 0.06. This low stress value suggests a high degree of accuracy in the two-dimensional representation of the fungal community data (Figure 3B). The observed differences were statistically significant (*p* < 0.00001), as confirmed by PERMANOVA tests, underscoring the substantial variation in fungal community structures between the conducive and suppressive soils (Figure 3B). The Shannon diversity index increased by 35% in the low Fusarium wilt incidence field, F4, highlighting the biological relevance of the diversity differences between suppressive and conducive soils. Both PCoA and NMDS analyses indicated that the low Fusraium wilt incidence field, F4, was distinct and separated from the other investigated fields, underscoring the unique fungal ecology of field F4 (Figure 3B). The distinct separation suggests that field F4 harbors fungal taxa potentially responsible for pathogen suppression, warranting further exploration of these taxa’s functional roles. Additionally, the high Fusarium wilt incidence fields F1, F2, and F5 were closer to each other compared to the low Fusarium wilt incidence field F3 (Figures 3A,B). The NMDS phylum-level composition analysis revealed the distribution of the most abundant fungal phyla across the different conducive and suppressive fields (Figure 3C). In the low Fusarium wilt incidence F4, phyla such as *Ascomycota* and *Basidiomycota* showed distinct clustering, indicating a unique community structure compared to the other fields. The high Fusarium wilt incidence fields F1, F2, and F5, on the other hand, displayed a closer relationship in the composition of their dominant fungal phyla, such as *Mucoromycota* and *Mortierellomycota* (Figure 3C). Hierarchical clustering provides a dendrogram view of the relationships among fungal communities across all fields. Additionally, all samples from the low Fusarium wilt incidence F4 (F4.1, F4.2, F4.3, F4.4) form a distinct cluster, reinforcing the uniqueness of its fungal community structure (Figure 2D). This is consistent with the PCoA and NMDS results, which also indicated a distinct separation of F4 from other fields (Figure 3D). The high Fusarium wilt incidence fields F1, F2, and F5 display varying degrees of clustering, with F2 forming a separate cluster, while F1, F3, and F5 cluster more closely together (Figure 3D).

### 3.3 Distinct taxonomic profiles highlight diversity in fungal communities across high Fusarium wilt and low Fusarium wilt incidence soils

**Figure 4:**
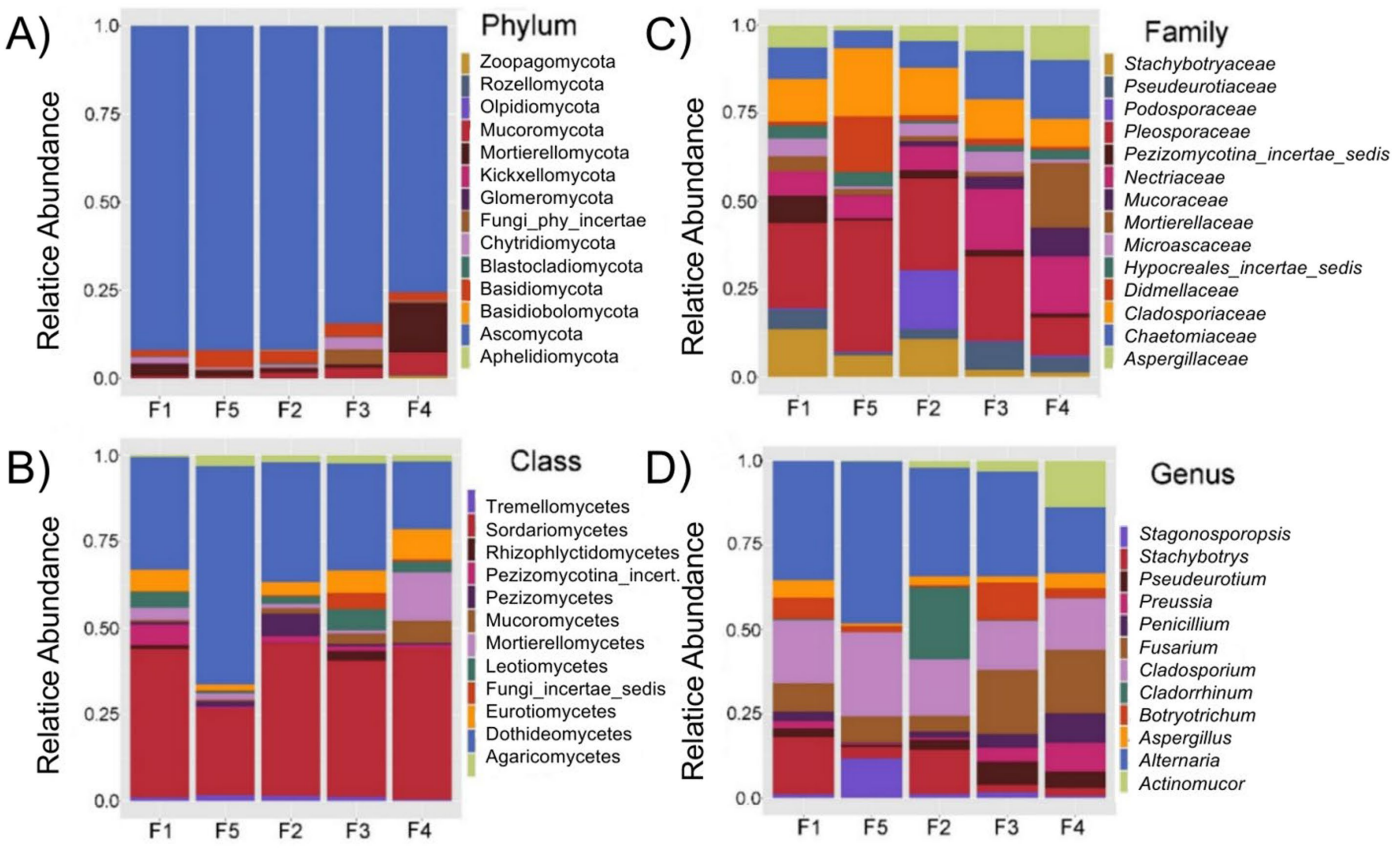
Taxonomic composition of fungal communities across different conducive and suppressive fields. Taxonomy bar shows the relative abundance of the fungi community at **(A)** phylum, **(B)** class, **(C)** family, and **(D)** genus levels.

To investigate the key taxa associated with the distinct fungal communities in conducive and suppressive soils, we analyzed the relative abundance of different taxa at the phylum, family, class, and genus levels (Figures 4A–D). The comprehensive analysis of fungal community composition across the studied fields revealed substantial differences in the relative abundance of fungal taxa at the phylum, family, class, and genus levels, corresponding to fields with low Fusarium wilt incidence, fields F3 and F4, and high Fusarium wilt incidence fields F1, F2, and F5 (Figures 4A–D). At the phylum level, *Ascomycota* emerged as the most dominant phylum across all fields; however, its relative abundance was notably lower in the low Fusarium wilt incidence fields (F3 and F4), particularly in F4, where higher proportions of *Mucoromycota* and *Mortierellomycota* phyla were observed (Figure 4A). This indicates a more diverse fungal community in these fields compared to the high Fusarium wilt incidence fields F1, F2, and F5, which displayed a higher relative abundance of *Ascomycota* and lower proportions of other phyla, suggesting reduced fungal diversity (Figure 4A). This pattern of diversity was further confirmed at the family level, where the low Fusarium wilt incidence field F4 exhibited a rich composition of fungal families, including *Aspergillaceae*, *Chaetomiaceae, Mortierellaceae*, *Mucoraceae*, *Nectriaceae*, and *Podosporaceae*, aligning with the elevated Shannon diversity index and beta-diversity analysis results (Figure 4B). Conversely, the high Fusarium wilt incidence fields (F1, F2, and F5) showed a dominance of *Cladosporiaceae*, *Pleosporaceae*, and *Stachybotryaceae*, families that were comparatively less abundant in F4 (Figure 4B). These findings underscore the distinctive fungal profiles associated with varying levels of Fusarium wilt incidence. The class-level analysis further supported these observations, highlighting an increased representation of Mucoromycetes and Mortierellomycetes in low Fusarium wilt incidence fields, which reinforces the notion that greater fungal diversity contributes to decreased disease incidence (Figure 4C). At the genus level, the differences became more pronounced, with *Actinomucor*, *Fusarium*, *Penicillium*, *Preussia*, and *Pseudeurotium* genera being more abundant in the low FOV4 field F4, indicating potential roles in disease suppression or soil health promotion (Figure 4D). In contrast, *Alternaria*, *Cladosporium*, and *Stachybotrys* were more prevalent in high Fusarium wilt incidence fields, suggesting potential associations with increased disease prevalence (Figure 4D). The distinct fungal community composition observed in low FOV4 fields, particularly the enrichment of genera such as *Actinomucor* and *Penicillium*, suggests the potential of these microbes in enhancing soil health and suppressing *Fusarium* wilt.

### 3.4 Elemental composition analysis of suppressive and conducive soils from El Paso field

**Figure 5:**
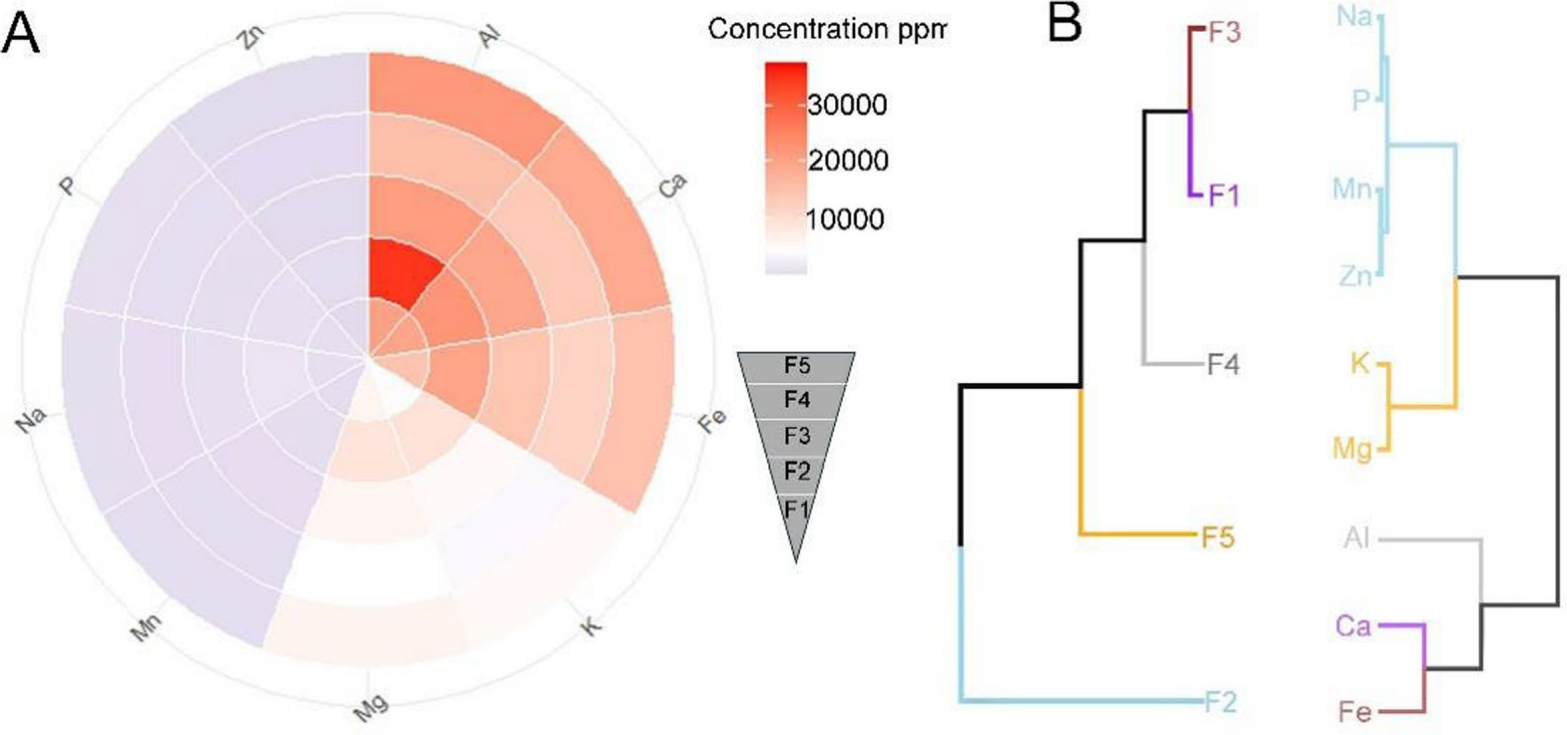
Elemental composition of soil samples from El Paso field. **(A)** Circular heatmap displaying the concentration of various elements (Al, Ca, Fe, K, Mg, Mn, Na, P, Zn) in soil samples from five different fields (F1–F5). The color gradient from white to red indicates increasing concentration levels in parts per million (ppm). **(B)** Dendrogram illustrating the hierarchical clustering of elements based on their concentration profiles across the fields.

In this study, we also analyzed the elemental composition of soil samples from different suppressive and conducive fields within El Paso County to gain insights into the environmental factors that may influence microbiome diversity and functionality (Figures 5A,B). The circular heatmap (Figure 5A) reveals distinct differences in the concentration of elements across the five fields. Field F2, for instance, exhibits the highest concentrations of most elements, particularly aluminum (Al), calcium (Ca), and iron (Fe). Field F5 also shows relatively high concentrations of these elements, though not as pronounced as in F2 (Figure 5A). On the other hand, F4 generally has lower concentrations of the elements, which may suggest lower soil fertility. Additionally, fields F1 and F3 have moderate concentrations of elements, falling between the high levels in F2 and F5, and the low levels in F4 (Figure 5A). The dendrogram shows the hierarchical clustering of elements based on their concentrations across the fields, revealing three distinct groups. Group 1 includes sodium (Na), phosphorus (P), manganese (Mn), and zinc (Zn), all of which exhibited lower concentrations across all investigated fields (Figure 5B). Group 2 consists of potassium (K) and magnesium (Mg), both of which display intermediate concentrations across the fields (Figure 5B). Group 3 comprises Al, Ca, and Fe, which exhibited higher concentrations in fields F2 and F5 (Figure 5B). The dendrogram provides a hierarchical clustering of the fields based on their elemental composition, revealing three distinct groupings that reflect similarities and differences in soil properties (Figure 5B). Fields F2 and F5 grouped together in cluster I, indicating similar elemental compositions characterized by higher concentrations of Al, Ca, and Fe (Figure 5B). Fields F1 and F3 form a separate cluster II, exhibiting intermediate levels of elements such as K and Mg, which indicate moderate soil fertility. Cluster III contains Field F4, which is characterized by generally lower concentrations of most elements (Figure 5B).

## 4 Discussion

This study examined five cotton fields with varying levels of *Fusarium oxysporum* f. sp. vasinfectum race 4 (FOV4) incidence in the El Paso, TX region. A key finding was higher fungal diversity in low-incidence fields compared to high-incidence fields. Increased fungal diversity has previously been associated with enhanced soil suppressiveness and improved resistance to soilborne pathogens, likely due to greater niche competition and more complex microbial interactions that limit pathogen establishment (van Elsas et al., 2012; Schlatter et al., 2017). Low-incidence fields also showed higher relative abundances of fungal taxa such as *Actinomucor*, *Penicillium*, *Preussia*, and *Pseudeurotium*, which have been previously reported in suppressive soils or associated with antagonistic activity against plant pathogens (Mendes et al., 2018; Alabouvette et al., 2009; Bonanomi et al., 2018). For example, *Penicillium* species are known producers of antifungal secondary metabolites that can inhibit *Fusarium* growth, while members of the Ascomycota such as *Preussia* have been associated with saprotrophic activity that contributes to organic matter decomposition and microbial competition in soil ecosystems.

The correlation between VRS scores and mortality rates supports the use of VRS assessment as a predictive tool for early disease detection and management (Ulloa et al., 2006, 2022, 2023). The distinct fungal patterns observed across fields further suggest that soil fungal composition may serve as an additional indicator of disease risk, consistent with previous studies showing that shifts in fungal community structure often precede visible disease outbreaks in agricultural systems (Berendsen et al., 2018; Cha et al., 2016). These findings reinforce the importance of integrated disease management strategies that combine soil health monitoring, deployment of resistant cultivars, and routine pathogen surveillance to enhance cotton resilience against FOV4.

The increased fungal diversity observed in low-incidence fields may contribute to soil suppressiveness through multiple mechanisms, including resource competition, antibiosis, and induced systemic resistance in plants. These mechanisms have been widely documented in suppressive soils where diverse microbial communities collectively reduce pathogen fitness (Weller et al., 2002; Mendes et al., 2011). The results from this study collectively suggest that fungal diversity and community composition are closely associated with Fusarium wilt incidence, where low-incidence fields support more diverse and potentially protective microbial assemblages. This supports the hypothesis that soil fungal diversity is a key component of disease suppressiveness in agroecosystems.

The observed reduced genetic diversity within *Fusarium* taxa in high-incidence fields may be explained by the genomic architecture of *Fusarium oxysporum*. Recent genomic studies of virulent strains such as FOV4 and FOV7 have highlighted the role of accessory chromosomes in pathogenicity, which can be transferred horizontally between strains (Ma et al., 2010; van Dam et al., 2017). These chromosomes often carry effector genes, including Secreted in Xylem (SIX) genes such as *SIX9*, which are highly conserved among virulent U.S. isolates (Jobe et al., 2024). Horizontal gene transfer of these accessory elements can facilitate rapid adaptation and competitive dominance of specific pathogenic lineages in monoculture systems with limited crop rotation (Vogan and Higgs, 2011). This may result in the reduced phylogenetic diversity observed in high-incidence soils, where a single or few highly adapted genotypes dominate the fungal community.

From a management perspective, restoring soil fungal diversity may represent a promising strategy to mitigate Fusarium wilt incidence in cotton. Organic matter (OM) amendments have been shown to enhance microbial diversity and stimulate beneficial microbial groups capable of suppressing soilborne pathogens (Bonanomi et al., 2018; Lazcano et al., 2013). By increasing carbon availability, OM can promote the growth of antagonistic and saprotrophic microorganisms, including taxa such as *Penicillium* and *Actinomucor*, which were enriched in low-incidence fields in this study. Increased fungal competition may reduce niche availability for pathogenic *Fusarium* strains, thereby limiting disease progression. However, the effectiveness of OM amendments is highly context-dependent, and excessive or poorly managed applications may lead to unintended shifts in microbial balance or nutrient leaching. Therefore, additional field-scale research is needed to optimize OM application rates and timing across cotton production systems to sustain beneficial microbial diversity while maintaining yield stability.

## 5 Conclusion

In summary, our findings provide evidence of an important role for soil fungal diversity in suppressing Fusarium wilt incidence. The higher fungal diversity and presence of potentially beneficial taxa in low-incidence fields suggest these microbial communities may inhibit pathogen establishment and proliferation. Conversely, the decreased diversity and prevalence of pathogenic genera in high-incidence fields may contribute to increased disease susceptibility. These insights have important implications for developing targeted soil management practices and breeding programs to enhance cotton resilience against FOV4.

## Data Availability

The raw sequencing data generated in this study have been deposited in the National Center for Biotechnology Information (NCBI) Sequence Read Archive (SRA) under BioProject accession number PRJNA1430756 and BioSample numbers SRR37502861- SRR37502880 (representing the five experimental field sites).

## Footnotes

1
www.Unite.ut.ee/

## Glossary

Al: Aluminum
ANOVA: Analysis of Variance
ASV: Amplicon Sequence Variant
Ca: Calcium
EF1-*α*: Translation elongation factor 1-alpha
Fe: Iron
FOV: *Fusarium oxysporum* f. sp. *vasinfectum*
FOV1–FOV8: Specific races of the *Fusarium oxysporum* f. sp. *vasinfectum* pathogen
FOV4: Fusarium wilt race 4
FW: Fusarium wilt
HGT: Horizontal gene transfer
ICP-OES: Inductively coupled plasma optical emission spectrometry
IGS: rDNA intergenic spacer region
ITS: Internal transcribed spacer
K: Potassium
Mg: Magnesium
mg/kg: Milligrams per kilogram
Mn: Manganese
Na: Sodium
NMDS: Non-metric multidimensional scaling
OM: Organic matter
P: Phosphorus
PCoA: Principal coordinate analysis
PCR: Polymerase Chain Reaction
PERMANOVA: Permutational multivariate analysis of variance
ppm: Parts per million
qPCR: Quantitative Polymerase Chain Reaction
QIIME2: Quantitative Insights Into Microbial Ecology 2
SIX: Secreted in xylem (effectors)
VRS: Vascular root staining score
Zn: Zinc
